# Artificial Intelligence in Bone Metastases: An MRI and CT Imaging Review

**DOI:** 10.3390/ijerph19031880

**Published:** 2022-02-08

**Authors:** Eliodoro Faiella, Domiziana Santucci, Alessandro Calabrese, Fabrizio Russo, Gianluca Vadalà, Bruno Beomonte Zobel, Paolo Soda, Giulio Iannello, Carlo de Felice, Vincenzo Denaro

**Affiliations:** 1 Department of Radiology, University of Rome “Campus Bio-Medico”, Via Alvaro del Portillo, 00128 Roma, Italy; e.faiella@unicampus.it (E.F.); d.santucci@unicampus.it (D.S.); b.zobel@unicampus.it (B.B.Z.); 2 Department of Radiology, University of Rome “Sapienza”, Viale del Policlinico, 00161 Roma, Italy; c.df@uniroma1.it; 3 Department of Orthopaedic and Trauma Surgery, University of Rome “Campus Bio-Medico”, Via Alvaro del Portillo, 00128 Roma, Italy; f.russo@unicampus.it (F.R.); g.vadala@unicampus.it (G.V.); v.denaro@unicampus.it (V.D.); 4 Unit of Computer Systems and Bioinformatics, Department of Engineering, University of Rome “Campus Bio-Medico”, Via Alvaro del Portillo, 00128 Roma, Italy; p.soda@unicampus.it (P.S.); g.iannello@unicampus.it (G.I.)

**Keywords:** MRI, CT, bone metastasis, bone cancer, lung cancer, prostate cancer, machine learning, radiomics, signature

## Abstract

(1) Background: The purpose of this review is to study the role of radiomics as a supporting tool in predicting bone disease status, differentiating benign from malignant bone lesions, and characterizing malignant bone lesions. (2) Methods: Two reviewers conducted the literature search independently. Thirteen articles on radiomics as a decision support tool for bone lesions were selected. The quality of the methodology was evaluated according to the radiomics quality score (RQS). (3) Results: All studies were published between 2018 and 2021 and were retrospective in design. Eleven (85%) studies were MRI-based, and two (15%) were CT-based. The sample size was <200 patients for all studies. There is significant heterogeneity in the literature, as evidenced by the relatively low RQS value (average score = 22.6%). There is not a homogeneous protocol used for MRI sequences among the different studies, although the highest predictive ability was always obtained in T2W-FS. Six articles (46%) reported on the potential application of the model in a clinical setting with a decision curve analysis (DCA). (4) Conclusions: Despite the variability in the radiomics method application, the similarity of results and conclusions observed is encouraging. Substantial limits were found; prospective and multicentric studies are needed to affirm the role of radiomics as a supporting tool.

## 1. Introduction

Bone is the third most frequent site for metastatic localization, after lung and liver [1], with breast and prostate cancer accounting for almost 70% of primary tumors [2]. In most cases, bone metastases influence a patient’s short-term prognosis, as bone lesions can rarely be completely eradicated. Patients with bone metastases have the option of undergoing palliative care to reduce the size of the lesions, slow their growth, or allow for improvement in symptoms. Bone metastases lead to a sharp reduction in life expectancy: average survival in patients with bone metastases from melanoma is 6 months; from breast cancer, 19–25 months; and from prostate cancer, 53 months [3]. 

The improvement of therapeutic strategies to deal with the various forms of cancer has led to an increase in life expectancy and, consequently, a lengthening of the time a patient can coexist with metastatic disease [4]. The most frequent site of bone metastasis is the axial skeleton because of its high red marrow content [1,2,5,6], which is therefore frequently responsible for the increased morbidity and decreased quality of life of patients. 

The spectrum of clinical manifestations is very heterogeneous, ranging from complete absence of symptoms to severe pain, reduced mobility, pathologic fractures, spinal cord compression, bone marrow aplasia, and hypercalcemia. Hypercalcemia is in turn responsible for constipation, polyuria, polydipsia, and fatigue [2,7]. In the final stages, hypercalcemia may lead to cardiac arrhythmias and acute renal failure [1].

Therefore, to identify a proper course of treatment, it is essential to differentiate metastatic lesions from any primary or benign lesions of the bone. In order to assess the patient’s prognosis and choose the most appropriate medical treatment according to their life expectancy, bone metastases should be diagnosed at the time of the diagnosis of the primary tumor: the aim is to reduce the incidence of complications and improve the quality of life. 

Bone scintigraphy, computed tomography (CT), magnetic resonance imaging (MRI), and positron emission tomography (PET) are all capable of assessing the presence of bone metastases [8]. The sensitivity and specificity of bone scintigraphy are 78% and 48%, respectively, but despite its relatively low specificity which may require further imaging examinations, it is still the most widely available technique and the most suggested by the guidelines for the study of bone disease. The CT exam, with a sensitivity and specificity of 74% and 56%, respectively, can be used as a guide during interventional diagnostic procedures. In addition, CT allows simultaneous evaluation of bone and systemic staging, reducing the burden of imaging for patients. MRI shows a sensitivity and specificity of 95% and 90%, respectively. It is a radiation-free technique and is considered the imaging modality of choice for assessing metastatic spread in the bone marrow. 18F FDG-PET (fluorodeoxyglucose) has a sensitivity and specificity of 98% and 56%, respectively: the sensitivity may vary among different histologies, as some well-differentiated tumors can go undetected because of their low metabolism [9].

Radiomics is an emerging branch of artificial intelligence (AI) that involves converting digital medical images that contain information related to tumor pathophysiology, also known as features, into measurable and quantifiable data. These data, combined with clinical and qualitative imaging-derived data, can improve medical decision making [10]. 

The field of radiomics is constantly and rapidly evolving. The purpose of AI is to aid the physician in the assessment of lesions beyond subjective visual interpretation in order to obtain additional information about tumor behavior and pathophysiology that is otherwise not inferable by the human eye with currently used techniques. 

As a topic of relatively recent emergence and application, there is considerable variability in the workflow that determines the results of radiomics-related studies. For traditional radiomics approaches, the workflow is divided into specific steps: data selection, medical imaging evaluation/segmentation, feature extraction, exploratory analysis, and modeling. The acquisition technical specifications and medical image reading modalities, the software and how the segmentation of the regions of interest (ROIs) is produced, the feature extraction, and the algorithm of the predictive model are all subject to numerous factors, making the research, and therefore the literature on it, highly heterogeneous. The radiomics quality score (RQS) was introduced in order to evaluate the past and future radiomics studies by achieving homogeneity in study reporting [11].

The purpose of this review is to investigate the potential role of radiomics as a decision-supporting tool in predicting bone disease status, differentiating benign from malignant bone lesions, and characterizing malignant lesions at the genetic level.

## 2. Materials and Methods

MEDLINE databases, such as PubMed and Web of Science, were employed for the research, using the following strings: ((“radiomics” OR “machine learning”) AND (metastases OR metastasis) AND (“bone” OR “spine” OR “spinal”)).

No limitations were applied to the search strategy. The following criteria were used for the inclusion of the studies: (a) imaging analysis involved only CT and MRI modalities; (b) the studies addressed the ability of radiomics to predict, diagnose, or characterize bone lesions; (c) the studies involved humans only; (d) the articles were accessible through our institution; and (e) the publications were in English. 

Case studies, abstracts, reviews, letters to editors, editorials, and commentaries were excluded. We completed the search by manually reviewing the bibliography of all selected articles. 

Two reviewers conducted the search, selected the studies, and extracted data from each study independently. From a total of 100 articles, 13 research articles were considered suitable and then collected. 

The quality of the methodology was assessed according to the RQS as described by Lambin et al. [11].

Each of the 16 criteria, covering individual aspects of the radiomics workflow, was assigned a different maximum score in relation to its importance. The absence of feature selection and validation results in a reduction in the final score by −3 and −5 points, respectively. The two reviewers assigned, in agreement, the RQS to the selected studies in absolute and percentage values (maximum value of 36, representing 100%).

The following data were extracted from each study: title, authors, year and journal of publication, study objective, study design (retrospective or prospective), number of patients, CT and MRI technical information, software used for segmentation and feature selection, number and type of radiomics features considered, algorithms used for classification, summary of results, and RQS.

## 3. Results

Our search found 13 publications on radiomics as a decision support tool regarding bone lesions. All studies were published between 2018 and 2021 and were retrospective in design. Study characteristics, as recorded by the reviewers, are shown in Table 1. 

Eleven (85%) studies were MRI-based, whereas two (15%) were CT-based. Four (30%) studies were focused on whether radiomics could predict epidermal growth factor receptor (EGFR) mutation in spinal metastases of primary lung adenocarcinoma. Three (23%) studied bone metastases from prostate cancer: two aimed to predict the presence of bone metastases from prostate cancer, one studied the prognostic role in terms of overall survival (OS) and cause-specific survival (CSS) of radiomics in prostate cancer patients with bone metastases. Four (30%) studies aimed to differentiate bone metastases from other pathological conditions: two studies evaluated the ability of radiomics to differentiate bone metastases from benign vertebral bone disease, and two studies evaluated the ability of radiomics to differentiate bone metastases from other pathological bone lesions. One (7%) study aimed to differentiate between metastatic and nonmetastatic vertebral bodies, and one aimed to differentiate between metastatic lesions in the spine originating from lung cancer and other nonpulmonary cancers. 

### 3.1. EGFR Mutation Prediction in Spinal Metastasis from Primary Lung Adenocarcinoma

Jiang et al. [12] analyzed MRI-based multiparametric radiomics for EGFR mutation prediction on T2-weighted (T2W), T2-weighted fat-saturated (T2W-FS), and T1-weighted (T1W) images: both traditional handcrafted and deep learning-based features were derived from each MRI sequence. For each of the two types of approach, radiomics models showed better results using combined features from all the MRI sequences than those with features extracted from each individual sequence. A fusion model created by integrating traditional handcrafted and deep learning-based features from the three sequences achieved the best prediction performance. A radiomics nomogram was obtained by integrating the best performing radiomics features: a decision curve analysis (DCA) confirmed the potential clinical utility of the radiomics nomogram.

Ren et al. [13] produced a nomogram using an MRI-based radiomics signature and smoking status to classify patients with EGFR mutation and wild-type EGFR through analysis of spinal metastases on T2W, T2W-FS, and T1W images. In addition to the radiomics model, a deep learning approach was considered: the combined signature generated higher AUCs than either feature type alone. Four different machine learning classifiers were developed and compared, with logistic regression outperforming the others. The nomogram achieved an AUC of 0.821 (SEN = 0.667, SPE = 0.909): DCA showed that the nomogram had a higher net benefit than all treatment and nontreatment strategies when the threshold was greater than 0.013.

Fan et al. [14] proposed a predictive model that could determine the presence of EGFR mutation in spinal metastasis subregions. Spinal metastases were divided into subregions based on patient- and population-level clustering: marginal, fragmentary, and internal subregions and the total tumor region. Radiomics features were extracted from the subregions’ T2W-FS and T1W images. For both sequences, the radiomics signature derived from the inner subregions outperformed other subregions or the entire tumor regions in terms of AUC: the multiregion radiomics signature derived from merging the inner subregion from T1W and T2W-FS MRI achieved the best detection capabilities. The results suggest that the inner region is biologically more aggressive than the others. 

Ran et al. [15] further investigated the predictive ability of the EGFR mutation in spinal metastases by constructing a radiomics model that could identify the mutation subtype in exon 19 and exon 21. The radiomics signature derived from the T2W-FS MRI consistently outperformed the T1W-derived signature in terms of AUC, ACC, sensitivity, and specificity. A nomogram model was constructed by incorporating the combined radiomic signature, age, and CEA level, achieving an AUC of 0.881 in the validation set: a decision curve analysis (DCA) confirmed that the model potentially guides individual treatments for patients with lung adenocarcinoma.

### 3.2. Bone Metastasis from Prostate Cancer

Wang et al. [16] determined that multiparametric prostate MRI predicted the presence/absence of bone metastasis in prostate cancer patients using radiomics features alone and combined with free PSA level and Gleason score. The combined MRI features derived from T2W and DCE showed higher prognostic performance than features derived from the single sequence and Gleason score. The radiomics MRI model combined with clinicopathological features (free PSA level, age, and Gleason score) yielded the highest AUC (AUC = 0.916), further improving predictive performance.

Hayakawa et al. [17] investigated the potential prognostic value of clinical risk factors (anamnestic and laboratory data and histological prostate cancer characteristics), imaging features, and radiomics of pelvic bone metastases in patients with newly diagnosed prostate cancer: patients were studied for OS and CSS. Only shape-based features were detected as risk factors for OS, and “maximum 2D diameter”, defined as the largest tumor surface dimension in the axial plane, was detected as a risk factor for OS after multivariate analysis (HR = 1.007). None of the radiomics features were detected as a risk factor for CSS in the uni- and multivariate analysis. After multivariate analysis, LDH, hemoglobin, and “maximum 2D diameter” were detected as risk factors for OS, whereas total Gleason score, LDH, and maximum 2D diameter were detected as a risk factors for CSS. Radiation therapy to the prostate gland and bone metastases did not significantly improve both OS and CSS.

Zhang et al. [18] established and validated a radiomics model that combined prostate multiparametric MRI-based radiomics signature and clinical risk factors to predict bone metastasis in patients with prostate cancer before treatment. The radiomics signature constructed from features extracted from DWI, T2W-FS, and DCE images showed good predictive efficiency. The nomogram, which incorporated the radiomics signature based on MRI and clinical risk factors, had an AUC of 0.92 in the validation set. DCA also demonstrated the clinical use of the radiomics model, which had better discriminatory efficiency than t-PSA or radiomics signature alone.

### 3.3. Differentiation of Bone Metastases from Other Bone Diseases

Sun et al. [19] proposed a CT-based nomogram able to distinguish between benign and malignant bone tumors. The nomogram, obtained by combining the radiomics signature and clinical model (consisting of demographics and CT characteristics), had higher diagnostic performance than the clinical model, but there was no statistical difference compared with the radiomics signature (AUC = 0.823 in the validation set). The DCA showed that the nomogram had higher diagnostic performance than the clinical model and achieved greater net clinical benefits than the clinical and radiomics signature models when considered alone.

Xiong et al. [20] evaluated the discrimination ability in T1W and T2W-FS MRI sequences between bone lesions from multiple myeloma and metastasis through several machine learning models: support vector machine (SVM), k-nearest neighbor (KNN), random forest (RF), artificial neural networks (ANNs), and naïve Bayes (NB). The ANN classifier from T2W images showed the best performance, both in differentiating myeloma from metastases and for classifying metastasis subtypes.

Yin et al. [21] developed and validated a multiparametric prostate MRI-based radiomics model to differentiate primary sacral chordoma, giant cell sacral tumor, and metastatic sacral tumor. Radiomics features extracted from the combined T2W-FS and CE T1W images exceeded those from the T2W-FS or T1W images alone, but T2W-FS outperformed T1W images. The highest radiomics model AUC was achieved when clinical and imaging data were combined.

Zhong et al. [22] proposed an MRI-based radiomics nomogram to differentiate cervical spine osteoradionecrosis from metastasis in patients with nasopharyngeal carcinoma after radiotherapy. The nomogram model demonstrated good calibration and discrimination, and DCA indicated that, if the threshold probability of a lesion for diagnosis as osteoradionecrosis is >12%, the radiomics nomogram adds net benefit when compared to either the treat-all-patients scheme or the treat-none scheme.

### 3.4. Other Studies

The study of Filograna et al. [23] is the only study that demonstrated the ability of radiomics-based MRI to differentiate between metastatic and nonmetastatic vertebral bodies in non-radiotherapy-treated cancer patients with metastatic bone marrow disease from primary tumors of different nature (three lung cancers, one prostate cancer, one esophageal cancer, one nasopharyngeal cancer, one hepatocarcinoma, and one breast cancer). Internal cross-validation showed an AUC of 0.814 for T1W images and 0.911 for T2W images. One histogram feature (minimum gray level) and one textural feature (joint variance of the gray level co-occurrence matrix) were found to be the best-fitting features in T1W and T2W images, respectively.

Lang et al. [24] aimed to differentiate metastatic spine cancer derived from primary lung cancer and other nonpulmonary cancers (breast, thyroid, prostate, liver, kidney) using an ROI-based model, radiomics, and deep learning. The accuracy of the radiomics model when histogram and texture features were combined was higher than that when histogram and texture features were evaluated alone. By increasing the number of features from three to five, the accuracy showed slightly higher values (from 0.68 to 0.71 in the histogram + texture model). The accuracy of the radiomics model was worse than that of the hot-spot ROI-based (ACC = 0.79) and deep learning (ACC = 0.71 ± 0.043 and 0.81 ± 0.034) methods.

### 3.5. RQS Assessment and Study Limitations

The average recorded RQS was 22.6% (0–38.8%). This low score confirms what has been reported in other reviews in the field of radiomics, representing a relatively low quality of research methodology [25,26,27,28,29,30]. None of the reviewed studies were prospective in design, no external validation on a dataset from another institution was performed, no cost-effectiveness of the clinical application of the radiomic models was reported, and no datasets were made publicly available (although four authors allowed access to the datasets upon request). Two articles (15%) did not perform any validation of their results. In only four (30%) of the articles, multiple segmentations were performed to assess the robustness of features to segmentation variabilities. The majority of articles (12/13, 92%) performed a feature reduction to decrease the risk of overfitting. Eight (61%) studies reported discrimination statistics (such as ROC curve and/or AUC), and six (46%) studies reported calibration statistics. Six articles (46%) reported on the potential application of the model in a clinical setting with a DCA.

## 4. Discussion

The application of radiomics in the diagnosis and characterization of bone lesions is recent and constantly evolving, as is the entire field of radiomics. The articles identified by our two researchers are few in number and were all published within the period between 2018 and 2021, with approximately 70% in the period immediately after 2020. Reflecting the relative freshness of this area of research, all studies are retrospective, performed at a single center, and with a small study population, ranging from 8 to 176 patients.

Radiomics can not only predict the presence of bone metastases and differentiate skeletal regions without lesions from those containing metastases, but its application is able to determine the primary tumor, differentiate metastases from other bone lesions (both benign and malignant), and predict mutation status (such as EGFR). Apart from MRI and FDG-PET, which have high predictive values, the other imaging methods have relatively low sensitivity and specificity values, although they are easily accessible and widespread [8,9]. Despite the predictive capabilities of the traditional imaging methods, there is some clinical information regarding bone metastases, including the genetic status or the primary tumor, that the naked eye is not able to perceive, due to similar clinical and imaging manifestations. Complete pathological confirmation and histological analysis are currently only possible by sampling through bone biopsy, which is associated with relatively high procedural risks (such as vertebral artery or spinal cord damage) [31]. Radiomics models, by inferring quantifiable data from the features, allow obtaining information that, once applied in the clinical setting, can be decisive for the specific therapeutic treatment choice. Because data are extracted from noninvasive methods, and in most cases radiation-free methods, radiomics is a further step towards the reduction in a patient diagnostic burden, and at the same time towards a patient-centered medicine. Some studies have also constructed nomograms in order to graphically represent the mathematical relationship between radiomics features and other prognostic factors, both clinical and diagnostic, in order to improve the clinical applicability of a field still difficult for nonexperts to interpret.

All articles included among their limitations the relatively small sample size (<200 patients), the single-center nature of the study, and the selection bias introduced by the retrospective design. Even in studies in which validation was performed on an internal dataset, the absence of external validation leads to reduced evidence of the possible clinical application of the research: multicenter studies are necessary to validate radiomics models and nomograms. Some articles complained about the tediousness of manual segmentation, which, in addition to being time-consuming, is not free of human error despite the option of multiple segmentation: the hope is that the spread of automatic, or semiautomatic, segmentation will speed up the process and further reduce the margin of error.

Our review confirms the considerable heterogeneity in current radiomics research, as evidenced by the relatively low RQS value obtained when analyzing the reviewed studies (22.6%). There is not a homogeneous protocol used for MRI sequences among the different studies, although the highest predictive ability was always obtained in T2W-FS. Wide variability also exists in the software used for image segmentation and feature extraction; the number and the type of features explored, with and without feature selection method application; and even the models used to classify the final features. All of these elements contribute to the reduced reproducibility of the results, even if none of them are considered integral to the RQS assessment. 

As described above, the most critical limitation concerns the small sample size, which leads to selection bias. A possible way to overcome this important limit is to increase the number of patients under investigation or to extend the research and results validation to other centers. In fact, it is well known that, after a first validation of a radiomic model, a subsequent path of validation through multicenter studies is necessary to allow radiomics to get closer and closer to widespread clinical applicability, even and especially through prospective studies.

This review has some important limitations. To our knowledge, no other review has exclusively investigated the role of radiomics in the analysis and prediction of bone metastases, particularly the spine localization. Even within the field of radiomics, this is a niche subfield, as is evident from the low number of studies analyzed. This novelty, in addition to the high variability of the included studies, both in methodology and in objectives, prevented us from pursuing a robust meta-analysis. We expect that as radiomics evolves and becomes more widespread, there will be an increase in the number of patients included and more extensive validation of existing datasets. Another critical issue at this early stage of research is the ability to share data across public datasets that have already been validated, as currently none of the papers publicly released their data. 

In addition, we have deliberately eliminated from the research the studies based on scintigraphy and PET (we have not detected studies that have used ultrasound) and papers in non-English language or not accessible from our institution, reducing the number of the articles included. Due to an implicit publication bias, most articles on this topic focus on the use of MRI. This implies that many other methods, on which there are no current studies, do not result in a significant contribution to research in the radiomics field, a phenomenon that introduces further bias into our review. Furthermore, at the time of publication, it is safe to assume that there are additional feature extraction software and classification models currently in development that we are unaware of in the literature, which are therefore protected from our review.

## 5. Conclusions

In spite of the variability in the radiomics method application, the similarity of results and conclusions observed is encouraging. Furthermore, all six studies that have measured the possible application of the radiomics model in the clinical setting through DCA have shown a net benefit compared to the use of the other strategies alone, confirming the promising role of radiomics in guiding the choice of treatments for individual cancer patients.

## Figures and Tables

**Table 1:** Characteristics of the selected radiomics studies.

Authors	Publication Year	Objective	Journal	Number of Patients	Imaging Modality	Segmentation	Technique Used for Feature Selection	Validation	Classification	Features	Best Results	Calibration Statistics	Decision Curve Analysis	RQS
Jiang X et al. [12]	2021	Detect EGFR mutation in spinal metastasis in patients with primary lung adenocarcinoma	*Journal of Magnetic Resonance Imaging*	97	3T MRI T1W, T2W, T2W-FS	Manual, ITK-SNAP	Mann–Whitney U-test, LASSO, 10-fold cross-validation	Y	Logistic regression models	Handcrafted features: first-order, shape- and size- based, texture, filtered features	Fusion features: AUC = 0.771, ACC = 0.550, SEN = 0.750, SPE = 0.875	Y	Y	10/36 = 27.7%
Ren M et al. [13]	2021	Detect EGFR mutation in spinal metastasis in patients with primary lung adenocarcinoma	*Medical Physics*	110	3T MRI T1W, T2W, T2W-FS	Manual, ITK-SNAP	Intraclass correlation coefficient (ICC) analysis, Mann–Whitney U, LASSO, 10-fold cross-validation	Y	Logistic regression, random forest, neural network, and support vector machine	First-order, shape-based, and texture (1967)	Fusion features: AUC = 0.803 (0.682–0.924), SEN = 0.700, SPE = 0.818; nomogram, AUC = 0.882 (0.695–0.974), ACC = 0.808, SEN = 0.846, SPE = 0.846	Y	Y	11/36 = 30.5%
Fan Y et al. [14]	2021	Detect EGFR mutation in spinal metastasis in patients with primary lung adenocarcinoma	*Physics in Medicine & Biology*	94	3T MRI, T1W, T2W-FS	Manual, ITK-SNAP	Mann–Whitney U-test, LASSO, 10-fold cross-validation	Y	Logistic regression models	First-order, shape- and size- based, texture, high-dimensional features (1595)	Multiregional radiomics signature: AUC = 0.777 (0.612–0.967), ACC = 0.688, SEN = 0.615, SPE = 0.947	N	N	8/36 = 22.2%
Ran C et al. [15]	2020	Detect EGFR mutation subtypes in exons 19 and 21 in spinal metastasis in patients with primary lung adenocarcinoma	*Academic Radiology*	76	3T MRI, T1W, T2W-FS	Manual	Mann–Whitney U, LASSO, 10-fold cross-validation	Y	Logistic regression models	First-order, shape-based, and texture (1967)	T1W: AUC = 0.728 (0.526 0.903), ACC = 0.692, SEN = 0.692, SPE = 0.769; T2W-FS: AUC = 0.852 (0.706 0.998), ACC = 0.731, SEN = 0.846, SPE = 0.769; nomogram, AUC = 0.821 (0.692–0.929), SEN = 0.667, SPE = 0.909	Y	Y	12/36 = 33.3%
Wang Y et al. [16]	2019	Pretreatment prediction of bone metastasis in patients with prostate cancer	*Magnetic Resonance Imaging*	176	3T MRI T2W, T1W DCE	Manual, IBEX	Linear regression, ridge regression, logistic regression models	Y	Linear regression, ridge regression, logistics regression models	Shape, intensity, intensity histogram, GLCM, gray-level run (976)	Combined T2W and DCE: AUC = 0.898 (0.833–0.937), ACC = 0.821, SEN = 0.647, SPE = 0.782	Y	N	8/36 = 22.2%
Hayakawa T et al. [17]	2020	Investigate the potential prognostic value of clinical risk factors, image features, and radiomics of pelvic bone metastasis in newly diagnosed prostate cancer patients	*Japanese Journal of Radiology*	69	CT	Manual, 3D Slicer	N	N	Not available	Shape-based, first-order statistics, texture (105)	Maximum 2D diameter and least axis were detected as risk factors for OS (HR 1.007 and 1.013, respectively)	N	N	0/36 = 0%
Zhang W et al. [18]	2020	Pretreatment prediction of bone metastasis in patients with prostate cancer	*BMC Medical Imaging*	116	3T MRI, T2W-FS, DWI, DCE T1W	Manual, AK software	ANOVA	Y	Logistic regression models	Not available (204)	AUC = 0.84	Y	Y	14/36 = 38.8%
Sun W et al. [19]	2021	Distinguish between benign and malignant bone tumors	*Cancer Imaging*	206	CT	Manual, ITK-SNAP	LASSO	Y	Logistic regression models	Shape, statistical, texture, wavelet (1130)	Radiomic model, AUC = 0.781 (0.643–0.918); nomogram, AUC = 0.917	Y	Y	12/36 = 33.3%
Xiong X et al. [20]	2021	Differentiating between multiple myeloma and different tumor metastasis lesions of the lumbar vertebra	*Frontiers in Oncology*	107	3T MRI, T1W, T2W-FS	Manual	LASSO, 10-fold cross-validation	Y	Support vector machine, k-nearest neighbor, random forest, artificial neural networks (ANNs), and naïve Bayes	Histogram features, GLCM, GRLM, and an autoregressive model (282)	Differentiating myeloma and metastasis, ANN T2W-FS: AUC = 0.815, SEN = 0.879, SPE = 0.790; differentiating myeloma and metastasis subtypes, ANN T2W-FS: AUC = 0.648, SEN = 0.714, SPE = 0.775	N	N	8/36 = 22.2%
Yin P et al. [21]	2018	Differentiation between primary sacral chordoma, sacral giant cell tumor, and sacral metastatic tumor	*Journal of Magnetic Resonance Imaging*	167	3T MRI, T2W-FS, T1W CE	Manual, ITK-SNAP	ANOVA, LASSO, Pearson correlation, random forest	Y	Random forest	Histogram features, form factor features, Haralick, GLCM features, RLM (385).	Combined T2W and T1W CE: AUC = 0.773, ACC = 0.711; T2W, AUC = 0.678, ACC = 0.541; T1W CE, AUC = 0.592, ACC = 0.568	Y	N	9/36 = 25%
Zhong X et al. [22]	2020	Differentiating of cervical spine osteoradionecrosis from metastasis after radiotherapy in nasopharyngeal carcinoma	*BMC Medical Imaging*	123	1.5 MRI, T1W CE	Manual, MaZda	Intraclass correlation coefficient (ICC) analysis, combination feature selection algorithm (combination of Fisher coefficient, classification error probability combined with average correlation coefficients, and mutual information), LASSO, 10-fold cross-validation	Y	Logistic regression models	Histogram, gray-level co-occurrence matrix, run-length matrix, absolute gradient, autoregressive model, and wavelet (279)	Nomogram: AUC = 0.720 (0.573–0.867), ACC = 0.723, SEN = 0.800, SPE = 0.640	Y	Y	11/36 = 30.5%
Filograna L et al. [23]	2019	Differentiate between metastatic and nonmetastatic vertebral bodies in patients with bone marrow metastatic disease	*La Radiologia Medica*	8	1.5 MRI, T1W, T2W-FS	Not available	Wilcoxon test	N	Logistic regression models	Statistical/ histogram, morphological, and textural features (89)	T1W: AUC = 0.814 (0.685–0.942); T2W: AUC = 0.911 (0.829–0.993)	N	N	2/36 = 5.5%
Lang N et al. [24]	2019	Differentiate metastatic cancer in the spine originated from lung cancer and other nonlung tumors	*Magnetic Resonance Imaging*	61	3T MRI DCE	Manual, automatic	Random forest algorithm	N	Logistic regression models	Texture, histogram (33 × 3 maps)	3 features, histogram + texture: ACC = 0.68; 5 features, histogram + texture: ACC = 0.71;	N	N	1/36 = 2.7%

## Data Availability

The study did not report any data.
